# Supplementary figures and images for: SaLSa: A Combinatory Approach of Semi-Automatic Labeling and Long Short-Term Memory to Classify Behavioral Syllables
Source: eNeuro. 2023 Dec 4;10(12):ENEURO.0201-23.2023. doi: 10.1523/ENEURO.0201-23.2023 (PMC10714892; doi:10.1523/ENEURO.0201-23.2023)

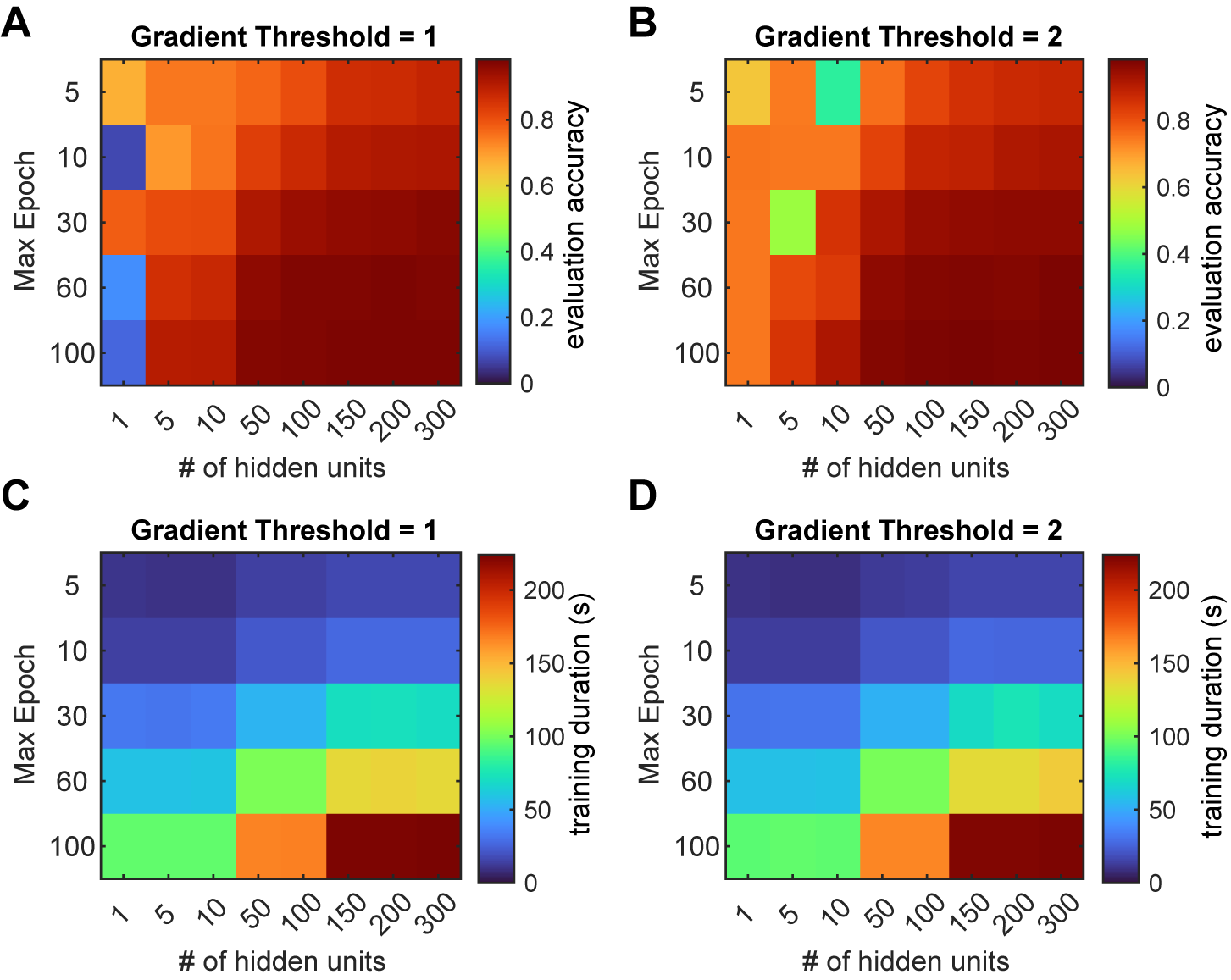

Supplement: Extended Data Figure 3-1 — Systematic comparison of three parameters for evaluation accuracy and training duration. A, B, The evaluation accuracy of LSTM models with variable maximum numbers of epochs and hidden units. The gradient threshold was set at 1 in A and 2 in B. C, D, Training duration across conditions. Download Figure 3-1, TIF file. [file enu-eN-MNT-0201-23-s02.tif]

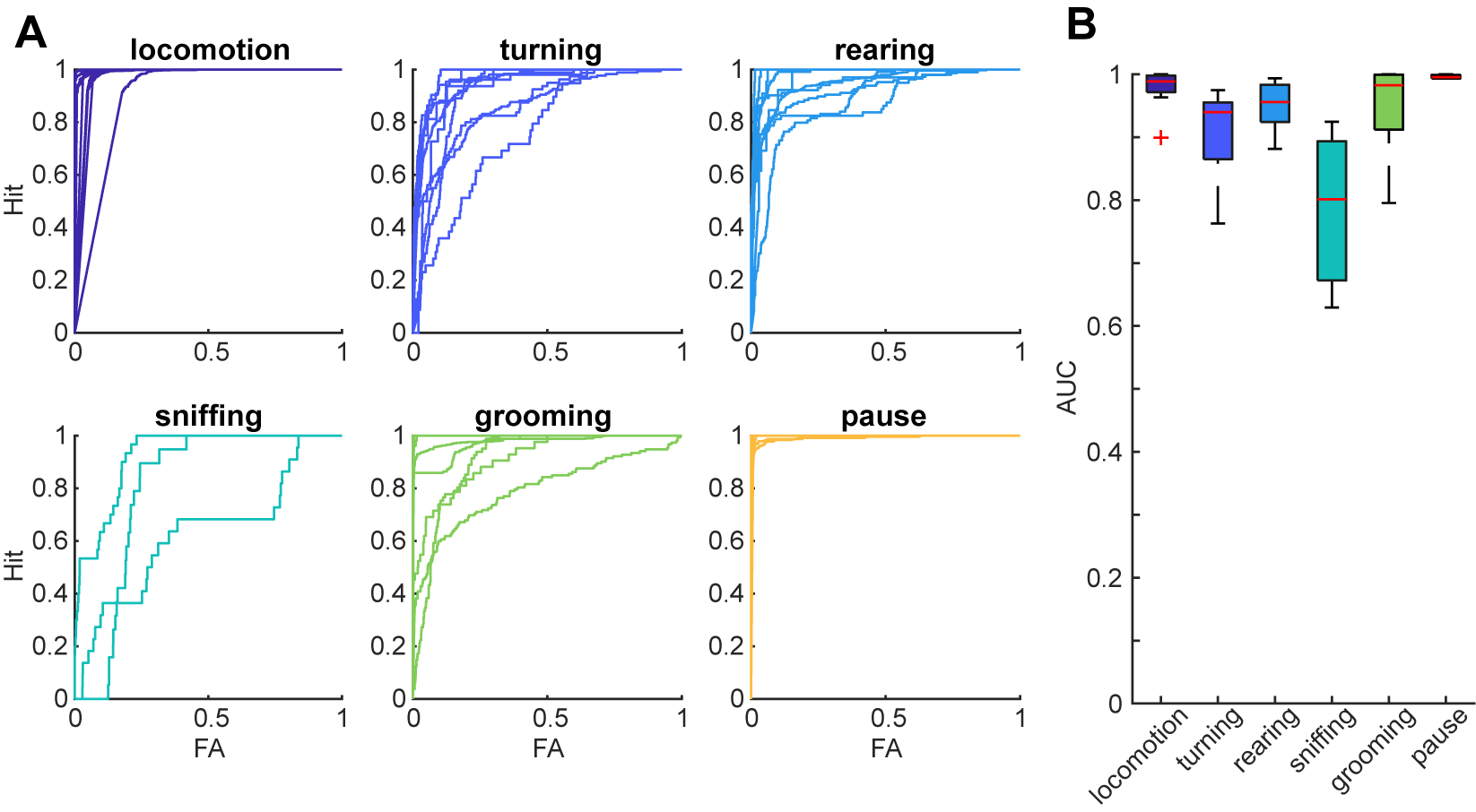

Supplement: Extended Data Figure 3-2 — Performance of a multiclass support vector machine. A, Receiver operating characteristic curves for each behavioral syllable based on independently labeled 10 videos. FA, false alarm. B, The area under the curve (AUC) values across behavioral syllables. Download Figure 3-2, TIF file. [file enu-eN-MNT-0201-23-s03.tif]
